# Agronomic responses and cattle performance in cultivars of tall fescue and orchardgrass under continuous stocking

**DOI:** 10.1093/tas/txaf120

**Published:** 2025-09-10

**Authors:** Kollin Frederick Johnson, Renata La Guardia Nave, Otávio Goulart de Almeida, Gary E Bates, Phillip Myer, Katie Mason

**Affiliations:** Department of Plant Sciences, University of Tennessee, Knoxville, TN, 37919, United States; Department of Plant Sciences, University of Tennessee, Knoxville, TN, 37919, United States; Department of Plant Sciences, University of Tennessee, Knoxville, TN, 37919, United States; Department of Plant Sciences, University of Tennessee, Knoxville, TN, 37919, United States; Department of Animal Science, University of Tennessee, Knoxville, TN, 37919, United States; Department of Animal Science, University of Tennessee, Knoxville, TN, 37919, United States

**Keywords:** animal daily gain, cool-season grasses, forage production, forage system, pasture

## Abstract

Tall fescue [*Schedonorus arundinaceus* (Shreb.) Dumort.; (TF)] is the primary forage species used by cow-calf producers in grazing systems in Tennessee. It is an excellent cool-season perennial grass due to its great forage mass (FM), nutritive value, and extensive growing season. Orchardgrass [*Dactylis glomerata* L.; (OG)] is also a widely used cool-season grass in the U.S. This study compared TF and OG cultivars under continuous stocking in terms of FM, nutritive value, and animal performance over two grazing seasons (2022 to 2023) in Spring Hill, Tennessee, U.S. Treatments were: 1) OG cv. Persist I (OG-P1), 2) OG cv. Persist II (OG-P2), 3) TF cv. Kentucky 31’ (TF-K31), and 4) a novel endophyte TF cv. Max Q (TF-NE). Forage mass was not affected by treatments (*P* = 0.0519), with an average of 2979 kg DM ha^−1^. Crude protein (CP) and neutral detergent fiber (NDF) concentrations were also unaffected by treatments (*P* = 0.6728, *P* = 0.1300), averaging 106 and 687 g kg^–1^, respectively. However, TF-NE had the lowest acid detergent fiber (ADF) (*P* = 0.0258; 380 g kg^–1^) and the greatest in vitro dry matter digestibility in 48 hours (INVDMD48) (*P* < 0.0001; 680 g kg^–1^), while OG-P2 had the greatest ADF (400 g kg^–1^) and the lowest INVDMD48 (661 g kg^–1^). Total gain and average daily gain (ADG) were affected by treatment × year interaction (*P* = 0.0314 and *P* = 0.0161, respectively). In 2022, TF-NE, TF-K31, and OG-P1 outperformed OG-P2, but no differences were observed in 2023 (total gain: 78.6 kg animal^–1^; ADG: 0.78 kg animal^–1^ day^–1^). Ergovaline concentrations in TF-K31 were low, which likely minimized its negative effects. These findings indicate that both TF and OG can sustain beef cattle production under continuous stocking, with TF offering potential advantages in botanical composition and forage nutritive value over time.

## Teaser

This study compared tall fescue and orchardgrass cultivars under continuous stocking to evaluate forage traits and cattle performance. A novel endophyte TF and an improved OG cultivar supported cattle gains similar to those of traditional TF and OG cultivars.

## INTRODUCTION

The transition zone of the United States (U.S.) lies between the cool-season and warm-season grass regions, where both types can thrive while still facing climatic challenges. However, tall fescue (*Schedonorus arundinaceus* [Schreb.] Dumort.; TF) dominates many cow-calf production systems in this zone due to its high forage yield, nutritive value, and persistence. The most common cultivar, Kentucky 31, owes much of its resilience and stress tolerance to a symbiotic relationship with an endophytic fungus (*Neotyphodium coenophialum*). While this endophyte enhances the plant’s resistance to drought and heat by producing alkaloids, it also generates ergot alkaloids, which are harmful to grazing livestock. These compounds are responsible for fescue toxicosis, a condition that significantly impairs reproductive and physiological performance in beef cattle, leading to estimated annual losses of up to two billion dollars in the U.S. beef industry (Aiken and Strickland, 2013).

In response to this issue, researchers have developed novel TF endophyte (NE) cultivars that retain the agronomic benefits of endophyte infection without producing toxic ergot alkaloids. Studies have shown that these NE varieties maintain similar levels of persistence and productivity compared to endophyte-infected (E+) TF, while significantly improving cattle performance (Hill et al., 1991; Hancock and Andrae, 2017). For example, grazing trials with stocker cattle demonstrated that NE TF can produce 135% more gain per hectare than E + fescue ( Parish, 2001). Despite these advantages, the high establishment costs and the need for proactive management, such as preventing contamination from surrounding E + stands, raise concerns about the long-term economic viability of NE TF systems (Gunter and Beck, 2004).

Orchardgrass (*Dactylis glomerata* L.; OG), another cool-season perennial forage species, is widely valued for its high palatability, forage quality, and suitability for both hay and grazing systems. Historically, OG has been viewed as a persistent species capable of withstanding multiple harvests annually while maintaining a stand longevity of 10 yr. However, recent concerns regarding its declining persistence under grazing and drought conditions have led to the development of improved, grazing-tolerant cultivars (Jones and Tracy, 2015). One such cultivar, “Persist,” was developed by the Tennessee Agricultural Experiment Station and released in 2000. It was bred from long-lived, regionally adapted OG ecotypes collected between 1959 and 1961 (Fribourg and Burns, 1961). In multi-location yield trials, Persist consistently produced equal or greater forage yields than other cultivars and demonstrated superior persistence under grazing pressure (Conger, 2003). After 4 yr of grazing with moderate drought stress, Persist maintained a 78% stand presence, while a competing cultivar, Benchmark, fell below 10% (Conger, 2003). Building on the success of Persist, breeders later developed “Persist II,” an OG cultivar with enhanced grazing tolerance. Using seed stock from Persist, new selections were subjected to intensive sheep grazing and evaluated for yield and persistence.

Despite these advancements in cultivar development, few studies have directly compared the performance and persistence of TF and OG under continuous grazing systems. Furthermore, the impact of these forages on cattle productivity, such as forage intake, conversion efficiency, and physiological responses, remains limited. The interaction between TF and clovers is particularly interesting, specifically red clover (*Trifolium pratense* L.) and white clover (*Trifolium repens* L.). These legumes are known to mitigate the effects of fescue toxicosis due to their phytoestrogenic compounds, such as isoflavones, which have been shown to influence vascular function and ruminal microbial communities (Flythe and Kagan, 2010; Melchior et al., 2018).

Given these factors, this study evaluated TF and OG under continuous stocking, mixed with red and white clover. The key objectives were to assess agronomic responses and cattle performance. This research aimed to identify the most viable grazing system for enhancing animal productivity and pasture sustainability in cow-calf operations within the U.S. transition zone.

## MATERIAL AND METHODS

### Site Description, Treatments, Experimental Design, and Measurements

The research was conducted at the University of Tennessee, Middle Tennessee AgResearch and Education Center (MTREC) in Spring Hill, TN, U.S. (35º 42’ N, 86º 56’ W, 482 m elevation). The experimental soil area is classified as Maury silt loam (Typic Paleudalf). Initial soil samples were collected to a depth of 15 cm and sent to the University of Tennessee Soil, Plant, and Pest Center laboratory in Nashville, Tennessee, for analysis (Hanlon and Savoy, 2007) (Table 1). Initial soil phosphorus (P) concentrations varied widely among paddocks (Table 1) due to differences in prior land use and nutrient management before study initiation. Based on the University of Tennessee fertility guidelines (Hanlon and Savoy, 2007), no P fertilizer was applied because all paddocks tested at or above the threshold for adequate establishment and growth of cool-season forages.

**Table 1.** Soil nutrients profile of the experimental site in Spring Hill, Tennessee, U.S.

Treatment	Replication	pH	P	K	Ca	Mg
	----------------------- kg ha^–1^ -----------------------					
OG-P1	1	6.1	64	61	1623	166
OG-P1	2	5.9	501	149	2601	177
OG-P1	3	5.7	482	136	2371	152
OG-P2	1	5.7	396	69	2186	169
OG-P2	2	6.0	946	68	3977	233
OG-P2	3	6.2	404	122	2467	244
TF-K31	1	6.1	74	46	1671	154
TF-K31	2	5.9	2712	119	7032	293
TF-K31	3	5.8	378	140	2471	212
TF-NE	1	5.9	111	72	1842	168
TF-NE	2	6.1	1015	113	4463	203
TF-NE	3	5.7	612	76	2900	210

Treatments = orchardgrass cv. Persist I (OG-P1), orchardgrass cv. Persist II (OG-P2), tall fescue cv. Kentucky 31’ (TF-K31), and novel endophyte tall fescue cv. Max Q (TF-NE).

Previously, the paddocks were composed of either switchgrass (*Panicum virgatum*) or TF. To prepare the paddocks for seeding, all switchgrass paddocks were cut for hay on 4 September 2020 and allowed to regrow for 2 wk. Existing TF paddocks were also clipped on 4 September 2020 and allowed to regrow for 2 wk. Subsequently, all 12 paddocks underwent an initial burndown spray treatment consisting of glyphosate (Cornerstone) (WinField United, Minneapolis, Minnesota, U.S.) applied at 2.8 L ha^−1^ plus surfactant at 1.9 L per 378 L ha^−1^ of water. The burndown application occurred on 21 and 22 September 2020.

Paddocks were seeded from 28 September 2020 to 1 October 2020 using a no-till drill (Great Plains, Salina, Kansas, U.S.). Orchardgrass varieties (Persist I and Persist II) were seeded at 17 kg ha^−1^, while the TF varieties were seeded at 22 kg ha^−1^. Following soil test recommendations, all 12 paddocks were fertilized on 7 October 2020 with approximately 33.6 kg ha^−1^ of nitrogen (N) and 100.8 kg ha^−1^ of potassium (K). Additionally, 4.49 Mg ha^−1^ of lime was applied to all paddocks to correct the soil pH.

Following the initial seeding, some preventive measures were implemented to control excessive weed growth, particularly wild barley (*Horduim vulgare* L.), which could shade out newly established forage stands. These measures included a spray application of 2,4-D to all paddocks on 9 February 2021 at a rate of 1.42 L ha^−1^ of 2,4-D (Shredder Amine) (WinField United, Minneapolis, Minnesota, U.S.) and 0.95 L ha^−1^ of surfactant per 378 L ha^−1^ of water.

On 15 March 2021, all paddocks were broadcast seeded with 2.2 kg ha^−1^ of white clover (*Trifolium repens* L., cv. Ladino; WC) and 2.2 kg ha^−1^ of red clover (*Trifolium pratense* L., cv. Rustler; RC) using a Herd ATV Seeder (Ford Distributing, Columbus, Ohio, U.S.). Shortly thereafter, 33.6 kg ha^−1^ of N and K was applied on 23 March 2021 to prepare the paddocks for spring growth.

Additionally, all paddocks were mowed to a height of 15 cm on 23 April 2021 and 23 June 2021. On 11 and 12 August 2021, eight of the twelve paddocks (those with preexisting switchgrass) were rope-wicked (Wick Wiper, Rosco, Lyon, Mississippi, U.S.) in both directions using glyphosate at a rate of 1.4 L ha^−1^ (50% of the initial burndown rate of 2.8 L ha^−1^), to control the heavy presence of switchgrass. Rope wicking is a process that utilizes a reservoir, typically a capped PVC pipe with ropes attached to the bottom, allowing herbicide in the reservoir to saturate the rope and selectively transfer the fluid to the plants it comes in contact with. Wicking was performed to selectively control the taller switchgrass without exposing the OG, TF, and clover to herbicide application.

On 9 September 2021, forage from all paddocks was harvested to remove excess forage and baled for hay. This harvest was conducted to facilitate uniform seeding across paddocks. Bale mass and the number of bales produced per paddock were recorded for internal management purposes only and were not analyzed as experimental variables. Following the hay harvest, on 11 and 12 October 2021, all paddocks were reseeded with OG or TF varieties with a no-till drill (Great Plains, Salina, Kansas, U.S.) at the same rates previously described to ensure uniform stand density and competitive balance among treatments, as establishment success was uneven following initial seeding. On the same dates, paddocks were also broadcast-seeded again with the 2.2 kg ha^−1^ WC and 4.4 kg ha^−1^ RC.

All paddocks were clipped to approximately 45 cm on 17 May 2022, the estimated height of weed seed heads. Clipping was chosen instead of chemical application due to the newly established clover stand. All treatments were fertilized with 146 kg ha^−1^ of urea on 16 August 2022 in preparation for fall grazing. All paddocks were clipped to a height of 30 cm from 26 to 28 October 2022 to remove any remaining seed heads and weeds. On 9 and 10 November 2022, all paddocks were sprayed with 2.4 L ha^−1^ of 2,4-D to aid in the control of springtime broadleaf weeds. This application likely impacted the remaining clover existing in the paddocks, but it was necessary to improve pasture condition and reduce weed competitiveness with the cool-season grasses. In preparation for the spring grazing season, approximately 146 kg ha^−1^ of urea was applied on 20 March 2023. The process was repeated for fall grazing with another application of 146 kg ha^−1^ of urea on 15 August 2023.

The study occurred over two consecutive years, from April to October 2022 and May to September 2023. The experimental design was a randomized complete block with three replications. The experimental area consisted of 12 paddocks, each measuring approximately 1.2 ha. The treatments consisted of: 1) orchardgrass cv. Persist I (OG-P1), 2) orchardgrass cv. Persist II (OG-P2), 3) tall fescue cv. Kentucky 31’ (TF-K31), 4) novel endophyte tall fescue cv. Max Q (TF-NE). All treatments included white clover (*Trifolium repens* L., cv. Ladino) and red clover (*Trifolium pratense* L., cv. Rustler).

All paddocks were grazed by stocker cattle under an approved protocol by the University of Tennessee Institutional Animal Care and Use Committee (IACUC), protocol number 2843-0621. Three tester steers were randomly assigned to each paddock, and an additional two put-and-take steers were added or removed as needed, to maintain forage mass (FM) above 2000 DM kg ha^−1^ and stubble height of 5 cm. The target stubble height of 5 cm represented a minimum residual threshold, not a constant average. Residuals were monitored visually, and forage mass, which was determined monthly using a 0.1m^2^ forage quadrat, served as a guide to ensure this target was met. Stocking rates ranged from 2.5 to 4.2 AU ha^−1^. Forage mass was

During year one, grazing began on 3 May 2022. All paddocks remained active until four of them fell below the 2000 kg ha^−1^ threshold and were terminated on 06 July 2022. The remaining eight paddocks were terminated on 22 July 2022 due to a severe drought and lack of FM, having fallen below a 1000 kg ha^−1^ threshold. Fall grazing resumed on 1 September 2022 and continued through 14 October 2022. The 2022 grazing season totaled 97 grazing days across spring and fall. Year two began on 9 May 2023 and concluded on 7 September 2023, totaling 121 grazing days.

The TF pastures composed of Kentuck-31 (TF-K31) were analyzed for ergovaline content. To obtain representative samples, twenty random sites were selected within each pasture, and a handful of TF-K31 was cut close to the ground using scissors. Plants were carefully collected, ensuring only TF was selected, excluding other grasses or weeds. Sampling followed the University of Kentucky Veterinary Diagnostic Lab (UKVDL) Guidelines for Tall Fescue Sample Collection for Ergovaline Analysis. In this study, three TF-K31paddocks were analyzed, with toxicology results of 130 ppb, < 100 ppb, and < 100 ppb. Two of the three paddocks had ergovaline concentrations below the minimum quantification threshold of 100 ppb.

### Measurements

To monitor total aboveground FM, all paddocks were sampled starting at the beginning of the grazing period and then monthly (on a 30-d cycle) during the growing season. Exact sampling dates were 27 April, 17 May, 23 June, 21 July, 31 August, 29 September, and 14 October in 2022, and 17 May, 14 June, 13 July, 10 August, and 7 September in 2023.

On each sampling date, 10 FM samples were randomly collected and hand-clipped from a 0.1 m^2^ area at a 5 cm stubble height in each paddock. Samples were immediately separated into morphological (leaves, stems, and dead material) and botanical (grass, legume, and weed) components. After separation, samples were dried in a forced-air oven (Thermo Scientific Convection Heratherm Oven) at 55 °C for 72 hours to a constant weight. Once dried, samples were weighed to determine the percentage of each botanical and morphological component. The 10 respective samples were recombined to maintain sample homogeneity and ground using a Wiley Mill (Thomas-Wiley Laboratory Mill Model 4, Arthur H. Thomas Co., Philadelphia, PA) to pass through a 2-mm screen; followed by a cyclone sample mill (Foss Cyclotec, Foss North America, Eden Prairie, Minnesota, U.S.) to pass through a 1-mm screen (McIntosh et al., 2022). Additional drying of the prepared sample in a forced air oven at 55 °C was performed to ensure consistent moisture content before scanning with a near-infrared spectrometer (NIRS), thereby reducing variability in predicted results across all samples (McIntosh et al., 2022).

The samples were scanned in small ring cups using NIRS technology (Unity Spectrastar XL-R instrument Unity Scientific, Milford, Massachusetts, U.S.) to predict crude protein (CP), neutral detergent fiber (NDF), and in vitro true dry matter digestibility in 48 hours (IVTDMD48). Equations for the forage nutritive value analyses were standardized and verified for accuracy with the 2021 Mixed Hay Equation developed, provided, and licensed by the NIRS Forage and Feed Consortium (NIRSC, Berea, Kentucky, U.S.). Global H and neighborhood statistical tests were used to assess prediction accuracy; all forage samples fit the equation (H < 3.0) and are reported accordingly (Murray and Cowe, 2004). Units of measurement for nutritive value analyses are presented at 100% dry matter (DM) basis across the entire dataset.

Steers were sourced from preconditioned steer sales with an average expected weight of 272 kg. Cattle were sourced 1 wk before the start of each grazing season to allow for environmental acclimation prior to trial initiation. Randomization was used to assign steers into grazer and study animal groups for each treatment. Steers were weighed every 14 d throughout the grazing period. Exact weighing dates were 13 May, 6 June, 24 June, 6 July, 22 July, 1 September, 15 September, and 14 October in 2022, and 9 May, 23 May, 15 June, 5 July, 10 August, 24 August, and 7 September in 2023. Weights were collected as single, unshrunk measurements taken immediately after cattle were gathered from the pastures. Animals were assessed for weight changes using a Tru-test digital animal scale and electronic identification system (Auckland, New Zealand) to calculate total gain and average daily gain (ADG). The total daily gain was calculated as the difference between the weight at the end of the last period and the weight at the beginning of the first period.

### Statistical Analysis

The data were analyzed using the PROC MIXED procedure in SAS version 9.4 (SAS Institute, Cary, NC, U.S.). The model included the effects of treatment, year, and their interaction as fixed effects. Block (replication) was considered a random effect (Littell et al. 2006). Akaike’s Information Criterion (AIC) was used to select the appropriate covariance structure (Wolfinger and O’Connell, 1993). Linear predictor and quantile-quantile plots of the residuals were used to verify the homogeneity of variance. Treatment means were calculated and compared using the probability of difference (PDIFF), based on Student’s test at a significance level of *P* < 0.05.

## RESULTS

### Weather

**Figure 1.**
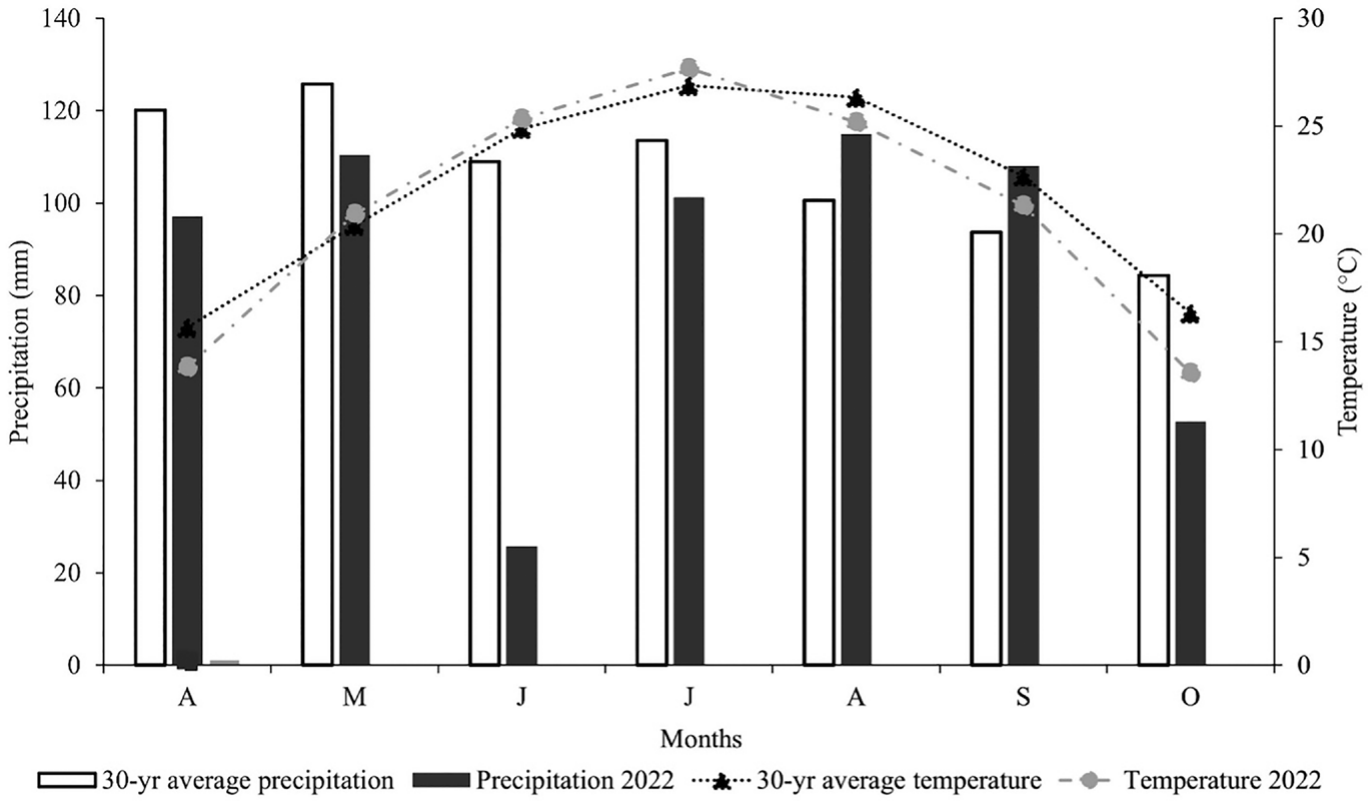
Weather for Spring Hill, TN, is reported as a monthly average based on daily records, including 30-yr averages for the grazing season of 2022.

In 2022, the mean air temperature from April through October was 21.1 °C, compared to the 30-yr running average of 21.9 °C, indicating a decrease of 0.8 °C (Figure 1). Rainfall showed a much more drastic deviation. While the 30-yr average precipitation was 106.7 mm, the mean rainfall in 2022 was only 87.1 mm. The most significant reduction occurred in June, with a 123.8% decrease in precipitation.

In contrast, in 2023, the average temperature from April through September was 21.8 °C, while the 30-yr average was 22.9 °C (1.1 °C warmer). Precipitation in 2023 was noticeably higher than the 30-yr average during July and August (Figure 1).

**Figure 2.**
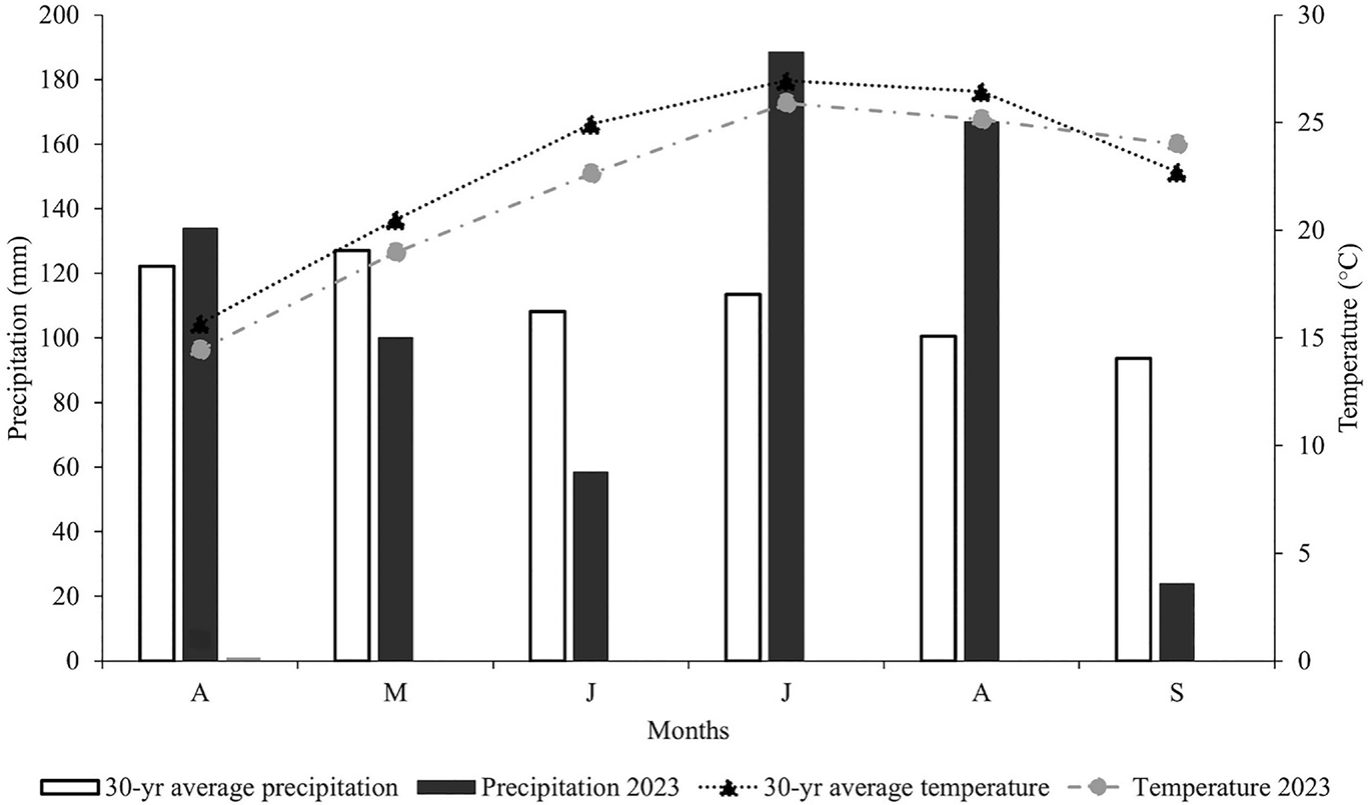
Weather for Spring Hill, TN, is reported as a monthly average based on daily records, including 30-yr averages for the grazing season of 2023.

Air temperatures in both years were considered adequate for forage growth. However, the sharp decline in rainfall during June 2022 and the increased precipitation during July-August 2023 created atypical growing conditions, which are believed to have influenced forage characteristics (Figure 2).

### Botanical and Morphological Composition

Leaf proportion was influenced by the treatment × year interaction (*P* = 0.0154) (Table 2). In 2022, there were no significant differences among treatments, with an average leaf proportion of 30%. However, in 2023, TF-K31 and TF-NE exhibited the highest leaf proportion, followed by OG-P1, while OG-P2 had the lowest. When comparing years, only OG-P2 was the only treatment that did not show a difference between 2022 and 2023, with an average value of 29%. In contrast, TF-K31, TF-NE, and OG-P1 showed increases in leaf proportion from 2022 to 2023 of 88%, 82%, and 60%, respectively (Table 2). Stem proportion was not affected by treatment, year, or the treatment × year interaction (*P* = 0.4890, *P* = 0.1303, and *P* = 0.8622, respectively), with an average 6% (SEM = 0.7).

**Table 2.** Leaf and weed composition in orchardgrass cv. Persist I (OG-P1), orchardgrass cv. Persist II (OG-P2), tall fescue cv. Kentucky 31’ (TF-K31), and novel endophyte tall fescue cv. Max Q (TF-NE) affected by treatment × year interaction during two grazing seasons, 2022 and 2023, in Spring Hill, Tennessee, U.S.

Treatment	Year		SEM^a^
	2022	2023	
	**---------- Leaf (%) ----------**		
OG-P1	30 Ab	48 Ba	3.9
OG-P2	25 Aa	33 Ca	3.9
TF-K31	33 Ab	62 Aa	3.9
TF-NE	33 Ab	60 Aa	3.9
SEM^a^	3.9	3.9	
	**---------- Weed (%) ----------**		
OG-P1	29 Aa	23 Ba	5.4
OG-P2	37 Aa	43 Aa	5.4
TF-K31	34 Aa	14 Bb	5.4
TF-NE	38 Aa	14 Bb	5.4
SEM^a^	5.4	5.4	

Means within a column without a common uppercase letter differ among treatments (*P* < 0.05).

Means within a row without a common lowercase letter differ among years (*P* < 0.05).

^a^SEM = Standard error of the mean.

The proportion of dead material was influenced by treatment (*P* < 0.0001) and year (*P* = 0.0030). The OG-P1 and OG-P2 had the highest proportions of dead material (33% and 29%, respectively), followed by TF-K31 (24%), while TF-NE had the lowest (19%) (SEM = 1.6). Across years, the proportion of dead material was greater in 2022 (26%) than in 2023 (18%) (SEM = 0.82).

Clover proportion was not influenced by treatment or treatment × year interaction (*P* = 0.4505 and *P* = 0.4505, respectively), with an overall average value of 1.3% across treatments. However, the year had a significant effect (*P* = 0.0005): in 2022, clover made up 2.6% of the botanical composition, while in 2023, it was absent.

Weed proportion was affected by treatment × year interaction (*P* = 0.0265) (Table 2). The weed community was dominated by wild barley (*Hordeum vulgare* L.), buttercup (*Ranunculus* spp.), spiny amaranth (*Amaranthus spinosus* L.), and common ragweed (*Ambrosia artemisiifolia* L.). In 2022, all treatments had a similar weed proportion of approximately 35%. In 2023, OG-P2 had the highest weed proportion, whereas OG-P1, TF-K31, and TF-NE showed no significant differences among them. When comparing years, OG-P1 and OG-P2 maintained similar weed proportions, while TF-K31 and TF-NE decreased by 59% and 63%, respectively (Table 2).

### Forage Mass

Forage mass was not affected by treatment (*P* = 0.0519), year (*P* = 0.7288), or the treatment × year interaction (*P* = 0.6173), with an overall average of 2970 kg DM ha^−1^ (SEM = 212.2).

During the first and second grazing periods in 2022, FM at both the beginning and end of each period did not differ among the treatments. Similarly, no differences were observed at the end of the grazing period in 2023. However, at the beginning of the 2023 grazing period, OG-P2 had the greatest FM, followed by OG-P1, while TF-NE had the lowest FM (Table 3).

**Table 3.** Forage mass available at the beginning and end of each grazing period in orchardgrass cv. Persist I (OG-P1), orchardgrass cv. Persist II (OG-P2), tall fescue cv. Kentucky 31’ (TF-K31), and novel endophyte tall fescue cv. Max Q (TF-NE) during two grazing seasons, 2022 and 2023, in Spring Hill, Tennessee, U.S.

Treatments	1° grazing period 2022		2° grazing period 2022		Grazing period 2023	
	Beginning	End	Beginning	End	Beginning	End
	**----------- Forage mass (kg DM ha** ^**–1**^ **) -----------**					
OG-P1	3524	2409	4168	2941	4835 AB	3700
OG-P2	3729	1748	4155	2904	4947 A	3219
TF-K31	3516	2793	3857	2778	4012 BC	3696
TF-NE	2593	1982	4506	2328	3634 C	3868
SEM^a^	561.1	371.1	266.3	246.4	350.3	499.1
*P*-value	0.5236	0.2947	0.1862	0.1188	0.0218	0.7145

Means within a column without a common uppercase letter differ among treatments (*P* < 0.05).

^a^SEM = Standard error of the mean.

The FM was affected by sampling time, with greater FM observed at the beginning compared to the end of each grazing period. Reductions in FM from the beginning to the end were 50%, 57% and 18% during the first and second grazing periods in 2022 and the single grazing period in 2023, respectively (Table 4).

**Table 4.** Average forage mass (FM) affected by beginning and end grazing period, 2022 and 2023, in Spring Hill, Tennessee, U.S.

Grazing period	1° grazing period 2022	2° grazing period 2022	Grazing period 2023
	**----------- Forage mass (kg DM ha** ^**–1**^ **) -----------**		
Beginning	3341 A	4171 A	4357 A
End	2233 B	2655 B	3696 B
SEM^a^	241.1	258.5	295.5
*P*-value	0.0032	< 0.0001	0.0367

Means within a column without a common uppercase letter differ among grazing period (*P* < 0.05).

^a^SEM = Standard error of them mean.

### Forage Nutritive Value

Crude protein concentration was not affected by treatment (*P* = 0.6728), year (*P* = 0.0586), or treatment × year interaction (*P* = 0.1870), with an overall average of 106 g kg^–1^ (SEM = 48). Neutral detergent fiber concentration was not influenced by treatment (*P* = 0.1300; with average value of 687 g kg^–1^, SEM = 54) or treatment × year interaction (*P* = 0.8025), but was affected by year (*P* < 0.0001), with a 6% increase in NDF observed from 2022 to 2023 (Table 5). Acid detergent fiber concentration was affected by both treatment and year (*P* = 0.0258 and *P* = 0.0239, respectively), but not by their interaction (*P* = 0.8025). The highest ADF concentration was observed in OG-P2, while the lowest was found in TF-NE (Table 6). Across years, ADF concentration increased by 3% from 2022 to 2023 (Table 5).

**Table 5.** Neutral detergent fiber, acid detergent fiber, and in vitro dry matter digestibility 48 hours concentrations affected by year, 2022 and 2023, in Spring Hill, Tennessee, U.S.

Year	Neutral detergent fiber	Acid detergent fiber	In vitro dry matter digestibility 48 hours
	----------- g kg^–1^ -----------		
2022	670	390	688
2023	710	400	655
SEM^a^	78	47	55

Means within a column without a common uppercase letter differ among grazing period (*P* < 0.05).

^a^SEM = Standard error of them mean.

**Table 6.** Acid detergent fiber and in vitro dry matter digestibility 48 hours concentrations affected by treatment, 2022 and 2023, in Spring Hill, Tennessee, U.S.

Treatment	Acid detergent fiber	In vitro dry matter digestibility 48 hours
	----------- g kg^–1^ -----------	
OG-P1	390 AB	665 AB
OG-P2	400 A	661 B
TF-K31	385 BC	676 A
TF-NE	380 C	680 A
SEM^a^	46	64

Means within a column without a common uppercase letter differ among grazing period (*P* < 0.05).

^a^SEM = Standard error of them mean.

The INVDMD48 was affected by treatment (*P* < 0.0001) and year (*P* = 0.0454), but not by the treatment × year interaction (*P* = 0.6776). The greatest INVDMD48 values were observed in TF-K31 and TF-NE, while OG-P2 showed the lowest digestibility (Table 6). Across years, INVDMD48 was 5% higher in 2022 compared to 2023 (Table 5).

### Animal Performance

Total weight gain and ADG were both influenced by the treatment × year interaction (*P* = 0.0314 and *P* = 0.0161, respectively). Across grazing periods, average stocking ranged from 2.5 to 4.2 AU ha-1, reflecting seasonal variation in forage mass. In 2022, the total gain was greater in TF-NE, TF-K31, and OG-P1, while OG-P2 exhibited the lowest gain. In contrast, no differences among treatments were observed in 2023, with an average total gain of 78.6 kg animal^–1^. When comparing years within each treatment, only OG-P2 showed an increase in total gain from 2022 to 2023; the other treatments did not exhibit a difference between years. In terms of ADG in 2022, OG-P2 had the lowest value compared to the other treatments. However, in 2023, no differences in ADG were observed among treatments, with an overall average of 0.78 kg animal^–1^ day^–1^. Across years, only TF-NE showed a reduction in ADG (29%), while the other treatments remained consistent (Table 7).

**Table 7.** Total gain and average daily gain in orchardgrass cv. Persist I (OG-P1), orchardgrass cv. Persist II (OG-P2), tall fescue cv. Kentucky 31’ (TF-K31), and novel endophyte tall fescue cv. Max Q (TF-NE) affected by treatment × year interaction during two grazing seasons, 2022 and 2023, in Spring Hill, Tennessee, U.S.

Treatment	Year		SEM^a^
	2022	2023	
	**------- Total gain (kg animal** ^**–1**^ **) -------**		
OG-P1	72.9 Aa	81.2 Aa	6.59
OG-P2	50.5 Bb	81.4 Aa	6.59
TF-K31	77.0 Aa	80.3 Aa	6.59
TF-NE	82.6 Aa	71.7 Aa	6.59
SEM^a^	6.59	6.59	
	**---- Average daily gain (kg animal** ^**–1**^ **day** ^**–1**^ **) ----**		
OG-P1	0.9 Aa	0.81 Aa	0.06
OG-P2	0.6 Ba	0.81 Aa	0.06
TF-K31	0.9 Aa	0.80 Aa	0.06
TF-NE	1.0 Aa	0.71 Ab	0.06
SEM^a^	0.06	0.06	

Means within a column without a common uppercase letter differ among treatments (*P* < 0.05).

Means within a row without a common lowercase letter differ among years (*P* < 0.05).

^a^SEM = Standard error of the mean.

## DISCUSSION

The structure of the sward can be characterized by its morphological and botanical composition, both of which are dynamic and responsive to plant growth and management (Allen et al. 2011 ; Almeida et al., 2023). Among these structural components, leaf proportion holds particular significance due to its central role in light interception (Vogelmann and Gorton, 2014) and as the primary site of nutrient intake for grazing animals (Moore et al., 2020). In the present study, TF-K31 and TF-NE exhibited a significant increase in leaf proportion from 2022 to 2023, surpassing 60% of the morphological composition, and outperforming both OG cultivars in this regard (Table 2). Leaf development is known to be plastic and responsive to environmental and management conditions, contributing to the observed seasonal variation (Insua et al., 2018). Supporting this leaf structure, the stem plays a vital role in maintaining canopy integrity and facilitating forage accessibility (Almeida et al., 2023).

In addition to its structural support function, the stem also plays a role in enhancing light penetration within the canopy, thereby promoting growth and supporting the development of new leaves at the upper strata of the sward (Lara et al., 2021). In this study, however, stem proportion did not differ among the cool-season grass cultivars and remained consistently low across treatments. Although excessive stem development can contribute to canopy shading and reduced forage quality, the limited stem growth observed here suggests it was not a major factor influencing the accumulation of dead material. Instead, the proportion of dead material appeared to be primarily associated with natural plant senescence, aging tillers, and environmental stressors such as drought or temperature fluctuations, which impair photosynthetic function and accelerate tissue degradation (Baron and Bélanger, 2020; Moore et al., 2020).

Beyond structural components, the botanical composition of pastures also includes species that enhance ecosystem function, such as legumes like RC and WC. These species contribute biologically fixed nitrogen to the system, offering an alternative to synthetic fertilizers (Collins et al., 2017; Corbin et al., 2024b). Although the integration of legumes is widely encouraged in forage systems, the clover proportion observed across both TF and OG cultivars in this study was very low, averaging only 1.3% of the total composition. This level is likely insufficient to meaningfully contribute to nitrogen input. To provide substantial nitrogen fixation (100 to 200 kg N ha^–1^ year^–1^), WC typically needs to comprise 20% to 30% of the DM in the sward (Ledgard and Steele, 1992). Moreover, Nyfeler et al. (2011) demonstrated that the most substantial forage production from grass-legume mixtures occurs under low to moderate N fertilizer (50 to 150 kg N ha^–1^ year^–1^) with legumes accounting for 40% to 60% of the canopy. Thus, the clover presence in this study was below the threshold needed for a measurable agronomic or ecological benefit.

Even in well-managed pastures, the weed proportion reflects the presence of plant species that compete with target forages, in this case, TF and OG cultivars. Weeds were defined in this study as any plant species other than the OG, TF, or clovers. The proportion of weeds declined in 2023 for both TF cultivars but remained unchanged for the OG cultivars. This reduction may be attributed to the competitive growth habit of TF (Nave et al., 2025), which can effectively suppress weed establishment by rapidly occupying canopy space and limiting light availability. Moreover, the growth advantage of TF and other cool-season grasses during spring and fall may have reduced the opportunity for C_4_ summer annual weeds to emerge and establish, as these species are typically dormant during cooler periods (Davies, 2001). The observed shifts in botanical composition, particularly the reduced weed pressure and increased presence of desirable species, likely contributed to maintaining or improving FM across the pasture system.

The FM was similar across all treatments, suggesting that, under the study’s environmental and management conditions, the cool-season grass cultivars, both TF and OG, showed comparable productive capacities over the two grazing seasons. One contributing factor may be the relatively low ergovaline concentrations observed in the TF-K31 pastures. Two of the three TF-K31 paddocks had ergovaline levels below or near the minimum quantification threshold (100 ppb), which likely mitigated the typical negative effects associated with toxic endophyte infection. This reduced toxicity potential may have contributed to more moderate growth behavior, allowing TF-K31 to perform similarly to novel endophyte TF and OG cultivars in terms of FM. Nonetheless, some differences emerged during the grazing periods. In particular, OG-P2 exhibited the greatest FM at the beginning of the 2023 grazing season, likely reflecting varietal differences in early spring regrowth potential. Orchardgrass is known for its early-season vigor, as it is among the first cool-season grasses to resume growth in response to rising soil temperatures and increasing day length in late winter (Silva et al., 2022). This rapid early growth provides a competitive advantage at the onset of the grazing season. In contrast, TF-NE showed the lowest FM at the beginning of 2023, which may be attributed to its slower regrowth following winter dormancy. However, the lack of differences among treatments at the end of the grazing periods suggests that compensatory growth and similar defoliation recovery from defoliation occurred across cultivars, leading to a convergence in FM by the end of each grazing cycle.

Across treatments and years, FM significantly declined from the beginning to the end of each grazing period. This reduction is expected and reflects the natural depletion of standing biomass due to animal consumption, trampling, and limited regrowth during active grazing. Both TF and OG are cool-season perennial grasses, with peak growth occurring from early spring through early summer (approximately February to June in the southeastern U.S.). During the summer months, elevated temperatures, moisture stress, and extended photoperiods suppress growth, leading to reduced FM (Henry et al., 2016; Quinby et al., 2021; Nassar et al., 2025). Notably, TF typically exhibits a secondary growth peak in the fall (September to November) due to its greater heat and drought tolerance compared to OG (Silva et al., 2022). These seasonal growth patterns explain the observed FM reductions during grazing, particularly when grazing occurs under suboptimal regrowth conditions in late spring and summer. While FM is essential for supporting grazing capacity, the forage nutritive value ultimately determines its potential to support animal performance.

Crude protein concentration averaged 106 g kg^–1^, which aligns with typical values for cool-season grasses grazed at a similar phenological state without supplemental N (Burns and Fisher, 2010). This concentration is adequate to meet the maintenance requirements of mature beef cattle (Harty and Olson, 2020). However, it is lower than the 131 g kg^–1^ reported by Nave et al. (2025) under similar conditions. The absence of a treatment effect on CP suggests that the cultivar differences had less influence than environmental conditions or management practices. Notably, the low clover contribution across both years, especially in 2023, when clover was absent, as well as the presence of weeds, likely limited the potential for enhanced CP concentrations.

The increase in NDF concentration from 2022 to 2023 suggests a shift toward more mature, fibrous forage, likely due to a higher proportion of grass in the sward. As grass dominance increases more rapidly than legume presence, NDF values typically rise, as reported by Zegler et al. (2018), Sleugh et al. (2000), and Zemenchik et al. (2002). In this study, the average NDF exceeded the values reported by Corbin et al. (2024b), where TF receiving 67 kg N ha^–1^ had an NDF concentration of 544 g kg^–1^. Elevated fiber concentrations are agronomically relevant, as NDF is a key determinant of dry matter intake (Allen et al., 2019), which may limit animal performance even under sufficient FM.

Nutritive value components vary in their cellular structure and digestibility (Wilson, 1994). In this study, OG-P2 had the highest ADF concentration and the lowest IVTDMD48, whereas TF-NE had the lowest ADF and the highest IVTDMD48. Despite this, the IVTDMD48 values for TF were lower than those previously reported by Corbin et al. (2024a) and Nave et al. (2025). These differences cannot be attributed to stem proportion, which averaged only 6%, but may instead reflect factors such as leaf age (Insua et al., 2018) or the presence of weeds (Table 2). This underscores the importance of considering the complete botanical composition when evaluating forage quality, as it directly influences nutritive value and, consequently, animal performance.

Animal performance did not vary much across treatments and years. While unshrunk weights may increase the variability due to gut fill, consistent timing and identical handling across treatments minimized systematic bias. The ADG and total gain remained relatively consistent across treatments. According to Cobin et al. (2024b), the TF fertilizer with urea (67 kg N ha^−1^), and supplemented with RC and sunn hemp (*Crotalaria juncea* L.), yielded average ADG and total gain values of 0.57 kg day^−1^ and 31 kg animal^−1^, respectively. Similarly, TF pastures mixed with alfalfa (*Medicago sativa* L.) or fertilized with N resulted in ADGs of 0.67 and 0.61 kg day^−1^, and total gains of 75.2 and 68.5 kg animal^−1^, respectively (Waldron et al., 2020). McLaren et al. (1983) studied various pasture mixtures, including TF-WC and OG-WC, showing ADGs of 0.52 and 0.82 kg day^−1^, and total gains of 58.0 and 100.0 kg animal^−1^, respectively. In comparison, in 2023, the ADG and total gain were approximately 0.78 and 76.6, respectively, which are within or above the ranges reported in previous studies.

While our results show similar animal performance across TF-NE, TF-K31, and OG treatments in the two grazing seasons of this study, this finding must be interpreted in the context of the measured ergovaline concentrations in the KY-31 paddocks. Two of the three TF-K31 paddocks had ergovaline < or ≈ 100 ppb (the minimum quantification threshold), and the third was 130 ppb. These relatively low concentrations are considerably below levels commonly associated with classic fescue toxicosis effects on growth and physiology reported in the literature (Gunter and Beck, 2004; Aiken and Strickland, 2013), which helps explain why KY-31 did not reduce animal performance in this study. Our data indicate that when ergovaline concentrations are low, TF-K31 can sustain competitive forage nutritive value and animal gains comparable to TF-NE cultivars and improved OG cultivars. Future studies that explicitly manipulate or stratify pastures by measured alkaloid concentration would be necessary to quantify the full extent of productivity differences attributable to endophyte toxicosis under realistic, variable field conditions.

These findings suggest that well-managed cool-season pasture, even with minimal clover presence, can support productive beef cattle growth under continuous stocking. Overall, these results indicate that although TF-NE showed a slight improvement in nutritive value, as measured by ADF and INVDMD48, animal performance remained similar throughout the study, and both grasses can be used under continuous stocking conditions.

## CONCLUSION

This 2-yr study demonstrated that TF-NE, TF-K31, and OG pastures managed under continuous stocking supported comparable FM, nutritive value, and animal performance. Botanical composition and weed competition differed among treatments, but clover persistence was low across all treatments. Importantly, ergovaline concentrations in TF-K31 were low, which likely reduced the negative effects typically associated with fescue toxicosis and contributed to similar animal performance across treatments. These findings suggested that under low-toxin conditions, TF-K31 can perform similarly to TF-NE or OG systems, while the improved OG-P2 showed competitive performance relative to OG-P1. Caution is warranted in extrapolating these results to pastures with higher ergovaline levels. Future studies should evaluate long-term productivity and persistence, explicitly stratify treatments by alkaloid concentration, and assess integrated weed and legume management strategies that can enhance system resilience.
